# Frailty trajectory over one year among residential aged care (nursing home) residents

**DOI:** 10.3389/fmed.2022.1010444

**Published:** 2022-11-03

**Authors:** Renly Lim, Thu-Lan Kelly, Andre Q. Andrade, Lisa M. Kalisch Ellett, Rebecca Bilton, Gereltuya Dorj, Nicole L. Pratt, Elizabeth E. Roughead

**Affiliations:** Quality Use of Medicines and Pharmacy Research Centre, UniSA Clinical and Health Sciences, University of South Australia, Adelaide, SA, Australia

**Keywords:** cognition, frail older adult, mortality, physical activity, nursing home

## Abstract

**Objectives:**

Large population-based studies examining frailty trajectory found a linear increase in frailty over time. The pattern in which frailty changes over time for an individual person is less well-described. We examined the frailty trajectory of older adults living in aged-care in Australia.

**Materials and methods:**

This secondary study used data from a randomised controlled trial involving 39 aged-care facilities in Australia. The trial intervention was an on-going pharmacist-led intervention occurring every 8 weeks over 12 months aimed at preventing medicine-induced deterioration and adverse reactions. Frailty was assessed using the Frailty Index. Participants were categorised as non-frail, pre-frail and frail. Individual frailty trajectory over 12 months was visualised using the alluvial plot. Case notes were examined to explore reasons for any rapid transitions in frailty status.

**Results:**

A total of 248 participants was included. At baseline, 40.3% were non-frail and 59.7% were pre-frail. The proportion of participants who were non-frail and pre-frail decreased over time; 15.7% were frail at 6 months and 23.4% were frail at 12 months. Overall, twenty different combinations of frailty transitions were identified over 12 months. Retrospective analysis of case notes suggest that death or transition from non-frail to frail was often preceded by hospitalisation, falls, medication change or clinically significant deterioration in grip strength or cognition.

**Conclusion:**

The degree of frailty increased over time, but there were variations in the individual trajectories. Regular monitoring of events that precede changes in frailty status is needed to identify strategies to prevent further deterioration in residents’ conditions.

## Introduction

Frailty is a state of increased vulnerability to external stressors due to a decline in reserve and function across multiple physiological systems (1, 2). The two most common operational definitions of frailty are the Frailty Index which is based on the deficit accumulation approach (3) and the frailty phenotype based primarily on physical components (4). Prevalence of frailty increases with age (5). A 2021 systematic review of 240 studies found the pooled prevalence of frailty was 11% for studies that included individuals age 50 years and above, while the pooled prevalence for studies in adults age 90 years and above was 51% (5). Frail individuals have an increased risk of adverse outcomes including mortality, hospitalisation, disability, cognitive impairment, falls and fractures, delirium, and nursing home admission (6–11). In Australia, up to one in two older adults are considered frail (11). The prevalence of frailty is expected to rise as the number of older adults in Australia continues to grow (12). In 2020, older people make up 16% of Australia’s population (12). It is estimated that by 2066, the proportion of older people in Australia will increase to about 23% (12).

The majority of population-based studies examining frailty trajectory found a linear increase in frailty over time (13–16). Studies that attempted to group frailty progression found that frailty trajectories can be broadly categorised into three types: rapidly increasing, moderately increasing, and stable frailty (14, 17). Individuals with rapidly increasing frailty were twice as likely to die within a year than those with stable frailty (14). What is less well described in the literature is the pattern in which frailty changes over time for an individual person and the frailty trajectory of older adults living in aged-care facilities. A 12-year longitudinal study in Europe reported that the rate of frailty fluctuations (i.e., up and down change in frailty) increases with age (16), meaning that the oldest in the population are likely to experience the most variations in their frailty trajectory.

In this descriptive study using data from a 12-month randomised controlled trial (18), this study aims to examine the frailty trajectory of 248 older adults living in residential aged care facilities in Australia. We graphically represent the frailty trajectory of each person according to trial arms, and explore the possible reasons for rapid changes in frailty (e.g., non-frail to death within 6 months) by examining individual case notes.

## Materials and methods

This paper describes a secondary study conducted using data from the Reducing Medicine-Induced Deterioration and Adverse Reactions (ReMInDAR) Trial (18, 19). The ReMInDAR trial was a multicentre, open-label, randomised controlled trial involving 39 aged care facilities in Australia occurring between August 2018 and June 2020. The Australian government subsidises a range of aged care services including care in the home, short-term care, and residential aged care (20). The term residential aged care in Australia is synonymous with nursing homes in the USA where care is provided 24 h a day, 7 days a week to relatively frail older residents.

### Participants

Residents were eligible to participate in the trial if they were taking at least four medicines or at least one medicine with anticholinergic or sedative properties. Residents were excluded if they were frail (>0.40 on the Frailty Index) (11, 21), had moderate or severe dementia [Psychogeriatric Assessment Scales < 12/21 (22) or Montreal Cognitive Assessment MoCA ≤ 17/30] (23), or were receiving palliative or respite care. The ReMInDAR trial (19) aimed to identify early onset of medicine-induced deterioration. For individuals who were already frail or with dementia, we considered it unlikely that the pharmacist intervention involving changes in medicines will have a significant impact on the person’s condition. Further, our study adopted an “opt out” approach which meant that residents needed to have the capacity to decline participation if they wish to do so. Participants who enrolled in the trial were included in this secondary study.

### Intervention

The trial intervention was an on-going pharmacist-led intervention occurring every 8 weeks over 12 months aimed at preventing medicine-induced deterioration and adverse reactions.

### Outcome

The trial primary outcome was change in Frailty Index from baseline to 12 months (11). The Frailty Index is a multi-dimensional assessment encompassing physical, medical, psychological, and social factors to measure frailty. The Frailty Index consists of 39 variables and ranges from 0 to 1 with 0 being the least frail.

The secondary outcomes were changes in cognition as measured using the Montreal Cognitive Assessment (23), 24-h movement behaviour measured using the GENEActiv accelerometer (24), grip strength measured using a handheld dynamometer, weight extracted from the resident serial weight chart, adverse events (e.g., falls, delirium, hospitalisation) and quality of life measured using the EQ-5D (25). Outcome assessments for all participants were conducted by independent research assistants at baseline, 6 and 12 months.

### Analysis

For the purpose of this secondary study, only the primary outcome (Frailty Index), which was of interest to understand frailty trajectory, was analysed. Participants were categorised at baseline as non-frail (Frailty Index < 0.25), pre-frail (≥0.25 to 0.4), and frail (>0.40) using previously validated Frailty Index cut-offs (21).

Descriptive statistics were used to summarise participants’ baseline characteristics. Baseline characteristics were presented as means (standard deviation) for continuous variables and as counts (percentage) for categorical variables. Individual frailty trajectory was visualised using the alluvial plot from the R package “ggalluvial.” Analyses were performed using the R statistical program, version 3.6.1 (R Foundation for Statistical Computing, Vienna, Austria) (26).

For participants who had a rapid progression in frailty (e.g., from non-frail to frail or non-frail to death within 6 months), we retrospectively examined individual case notes recorded by the trial pharmacists and research assistants to identify possible reasons for the change, and whether the pharmacists’ review and assessment using validated tools identified deterioration in participants’ health.

## Results

A total of 248 participants were included in this secondary study. The mean age of participants was 86 years and a third (32%) of the participants were men. The baseline characteristics of all participants included in this study is shown in Table 1.

**TABLE 1:** Baseline characteristics of all trial participants.

Baseline descriptor	*n* = 248
Age (years), mean (standard deviation, SD)	86 (7.41)
Gender = Male, *n* (%)	80 (32.3)
Weight, kg, overall, mean (SD)	73.60 (17.94)
Male weight, kg, mean (SD)	82.32 (16.08)
Female weight, kg, mean (SD)	69.45 (17.27)
Frailty Index, mean (SD)	0.27 (0.08)
Non-frail (Frailty Index < 0.25), *n* (%)	100 (40.3)
Highest Grip Strength, kg, mean (SD)	17.17 (7.42)
Grip Strength Male, kg, mean (SD)	23.02 (7.84)
Grip Strength Female, kg, mean (SD)	14.39 (5.29)
Montreal Cognitive Assessment (MoCA)*, mean (SD)	22 (3.38)
EQ-5D-5L single index, mean (SD)	0.67 (0.26)

**FIGURE 1:**
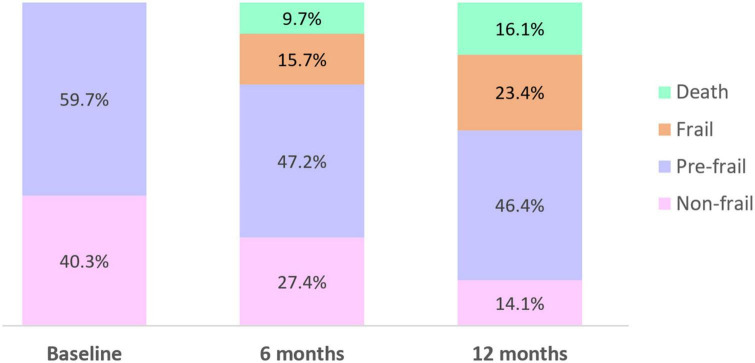
Proportion of participants who were non-frail, pre-frail, frail, or died over the 12-month study period.

At baseline, 40.3% of participants were non-frail (Frailty Index < 0.25) and 59.7% were pre-frail (0.25 ≥ Frailty Index ≤ 0.4) (Figure 1). No participants were frail at baseline, consistent with the trial exclusion criterion whereby residents with Frailty Index > 0.4 were excluded. The proportion of participants who were non-frail and pre-frail decreased over time; 15.7% of participants became frail at 6 months and 23.4% were frail at 12 months. Approximately 10% of participants died by 6 months and 16.1% by 12 months.

**FIGURE 2:**
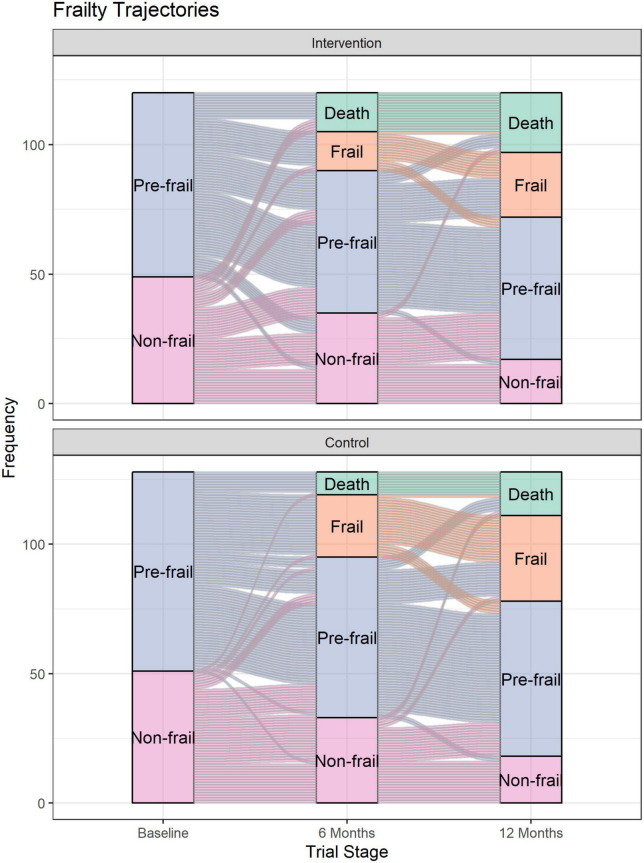
Frailty trajectory for intervention **(Top)** and control **(Bottom)** groups.

**FIGURE 3:**
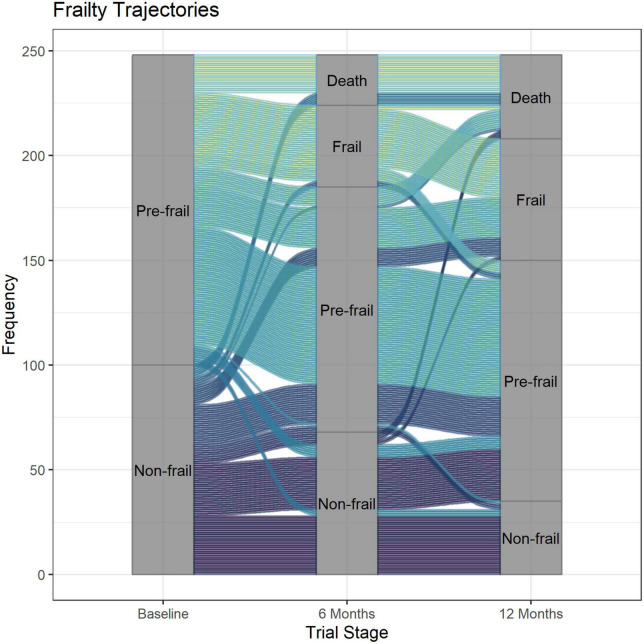
Frailty trajectory over 12 months for all study participants.

Figure 2 shows the individual trajectories for each participant from baseline to 12 months according to trial arm, while Figure 3 shows the trajectories of all participants from baseline to 12 months. There was an imbalance in the number of withdrawals due to death between the two trial groups. At the 6 months assessment, there were 15 (out of 120) and 9 (out of 128) deaths in the intervention and control groups, respectively. However, the imbalance in deaths predominantly occurred in the first 2 months, prior to the first intervention visit in the trial, with 5/120 deaths (4%) for intervention arm and 2/128 deaths (2%) for the control group.

Overall, twenty different combinations of frailty transitions were identified over 12 months (Table 2). The two common combinations of frailty transitions for non-frail participants were “stable frailty” (i.e., participants who were non-frail at baseline continued to be non-frail at 6- and 12-months) and “moderately increasing frailty” (i.e., participants moved from being non-frail to pre-frail at 6- or 12 months). The three common combinations for participants who were pre-frail at baseline were “stable frailty” (i.e., participants who were pre-frail at baseline and continued to be pre-frail at 6- and 12-months), “moderately increasing frailty” (i.e., moved from being pre-frail to frail at 6- or 12 months) and “pre-frail to death.” Ten percent (*n* = 10) of all participants who were non-frail at baseline died by 12 months, while 20% (*n* = 30) of all participants who were pre-frail at baseline died by 12 months.

**TABLE 2:** Frailty transitions identified over 12 months.

Trajectory	Baseline	Month 6	Month 12	*n* (%)
1	Non-frail	Non-frail	Non-frail	28 (11.3%)
2	Non-frail	Non-frail	Pre-frail	25 (10.1%)
3	Non-frail	Non-frail	Frail	2 (0.8%)
4	Non-frail	Non-frail	Death	4 (1.6%)
5	Non-frail	Pre-frail	Non-frail	3 (1.2%)
6	Non-frail	Pre-frail	Pre-frail	19 (7.7%)
7	Non-frail	Pre-frail	Frail	9 (3.6%)
8	Non-frail	Pre-frail	Death	1 (0.4%)
9	Non-frail	Frail	Pre-frail	3 (1.2%)
10	Non-frail	Death	Death	6 (2.4%)
11	Pre-frail	Non-frail	Non-frail	3 (1.2%)
12	Pre-frail	Non-frail	Pre-frail	6 (2.4%)
13	Pre-frail	Pre-frail	Non-frail	1 (0.4%)
14	Pre-frail	Pre-frail	Pre-frail	56 (22.6%)
15	Pre-frail	Pre-frail	Frail	19 (7.7%)
16	Pre-frail	Pre-frail	Death	9 (3.6%)
17	Pre-frail	Frail	Pre-frail	6 (2.4%)
18	Pre-frail	Frail	Frail	28 (11.3%)
19	Pre-frail	Frail	Death	2 (0.8%)
20	Pre-frail	Death	Death	18 (7.3%)

Twelve participants transitioned from non-frail to frail or death within a 6-month period. Retrospective analysis of case notes of the 12 participants suggest that death or transition from non-frail to frail was often preceded by hospitalisation, falls, medication change, or clinically significant deterioration as measured using grip strength or cognitive function test (Supplementary Table 1). Three participants who went from non-frail at baseline to frail at 6 months, transitioned back to pre-frail at 12 months (Supplementary Table 2). Similarly, the transition from non-frail to frail in the three participants were preceded by events such as falls, medication change or clinically significant deterioration in grip strength or cognition.

## Discussion

Our results showed that frailty increased over time as the proportion of residents who were pre-frail and frail increased over the 12-month study period, consistent with findings from previous research (13–16). A higher proportion of participants who were pre-frail at baseline died at 12 months compared to those who were non-frail at baseline. In contrast to only three trajectories that are commonly reported (14, 17), our study adds to the existing literature by graphically representing the individual trajectory of older adults living in residential aged care facilities. By visualising the individual trajectory, we were able to show that individuals who seemed “stable” (i.e., had similar baseline and 12-month Frailty Index) did actually experience transient changes in frailty in between follow-ups.

Although most residents either remained stable or had worsening frailty over time, our visualisation shows that a small number of residents had initial worsening of frailty but subsequently experienced improvement in frailty (e.g., non-frail at baseline, frail at 6 months and pre-frail at 12 months). This supports the notion that frailty can, to some degree, be reversed (16, 27–29). For example, a longitudinal cohort study involving 1,735 participants in five European countries found that participants who increased their physical activity over a 12-month period have lower frailty compared to baseline (28). By analysing individual case notes, we found that residents who had initial worsening frailty (at 6-month follow up) followed by improvement in frailty at 12 months experienced sudden stressors (falls and infection), and subsequent recovery leading to improved frailty. Frailty can also potentially be reversed by interventions to reduce frailty; however, previous systematic reviews found mixed results regarding the effectiveness of interventions to reduce or prevent frailty (27, 30). In our randomised controlled trial which involved a pharmacist-led intervention, we found no statistically significant difference in the change in Frailty Index between the intervention and the control groups (18). Our final sample for the trial was short of our required sample size of 354 persons, leaving the study under-powered for its primary endpoint (change in Frailty Index). The recruitment shortfall in the trial was in part due to the high proportion of residents in aged-care in Australia that are already frail. Despite using a Frailty Index cut off of 0.4, only 8% of the Australian aged-care population in the facilities recruited were eligible for our intervention.

One could argue that the within-individual change in frailty over time observed in our study was due to inter-operator variability in the assessment of Frailty Index, and not due to an actual change due to incidents with temporary harm such as infections and injury. However, we found similar fluctuations in scores measured using other validated tools (grip strength and cognition) for those in the intervention arm (19), suggesting that the within-individual changes in frailty are likely a true effect rather than inconsistency in measurement. Research has also shown that grip strength is correlated with frailty index (31), thus providing further support that that within-individual changes are a true effect.

To the best of our knowledge, this is the first study to visualise individual frailty trajectories and investigate reasons behind rapid changes in frailty among older adults living in aged care facilities (mean age 86). A recent study using the Irish Longitudinal Study of Ageing (TILDA) dataset similarly visualised frailty trajectories using the alluvial plot; however, the study included a younger cohort living in the community with a mean age of 63.8 years at baseline (32). The large number of frailty trajectories suggests that older adults in late life should be assessed regularly for signs and symptoms of deterioration, so that interventions can be implemented to prevent further deterioration. Interventions such as exercise and muscle strength training have been shown to be effective to delay or reverse frailty (33, 34). Our study had some limitations. We included a less frail population within the aged-care setting in our trial and therefore our results may not be generalisable to all aged care residents. The latter part of the trial (March to June 2020) was impacted by the COVID pandemic, which affected some of our 12 month data collection as our research assistants could not attend the facilities during that time. Frailty assessment was therefore delayed for a small number of participants.

## Conclusion

The degree of frailty increased over time in the aged care population, but there were variations in the individual trajectories experienced by residents. Regular monitoring of events that precede changes in frailty status is needed to identify strategies to prevent further deterioration in residents’ conditions. Future studies could consider visualising frailty trajectory for all residents and understanding the impact of COVID-19 including any restrictions due to the pandemic on frailty trajectories in aged care residents.

## Data availability statement

The data analysed in this study is subject to the following licences/restrictions: Requests for de-identified data can be made to the Principal Investigator. Requests to access these datasets should be directed to libby.roughead@unisa.edu.au.

## Ethics statement

The studies involving human participants were reviewed and approved by University of South Australia Human Research Ethics Committee. Written informed consent for participation was not required for this study in accordance with the national legislation and the institutional requirements.

## Author contributions

RL, T-LK, RB, and EER: acquisition, analysis or interpretation of data, and statistical analysis. RL: drafting of the manuscript. All authors contributed to the study concept and design and critical revision of the manuscript for important intellectual content, contributed to the article, and approved the submitted version.
