# Supplementary material for: Frailty trajectory over one year among residential aged care (nursing home) residents
Source: Front Med (Lausanne). 2022 Nov 3;9:1010444. doi: 10.3389/fmed.2022.1010444 (PMC9670112; doi:10.3389/fmed.2022.1010444)
Supplement: Supplementary file 1 [file Data_Sheet_1.pdf]

Table S1. Participants who transitioned from non-frail to frail or death within 6 months.

ID	Patient status at baseline, 6m and 12m	Trial arm	Primary reason of hospital admission	Death after admission	Grip strength	Cognition (MoCA)	Medication change
1	B: non frail<br>6m: Death	Intervention	Lower abdominal pain for investigation	One day after admission	B: 26.6<br>P1: 20.5*	B: 28	N/a
2	B: non frail<br>6m: death	Intervention	Pulmonary embolism	One day after admission	B: 11.8<br>P1: 13.1	B: 29<br>P1: 28	Sertraline (initiated two weeks before death)
3	B: non frail<br>6m: death	Intervention	Reason not known	Two days after admission	B: 17.1	B: 23	N/a
4	B: non frail<br>6m: death	Intervention	No admission recorded	N/a	B: 21.9<br>P1: 16.6*	B: 22<br>P1: 17*	
5	B: non frail<br>6m: death	Intervention	Deterioration	One day after admission	B: 34.3<br>P1: 35.8	B: 26<br>P1: 22*	Morphine, clonazepam, metoclopramide initiated in palliative care.<br>Note: All existing medications ceased.
6	B: non frail<br>6m: death	Control	No admission recorded	N/a	B: 26	B: 12.1	N/a
7	B: non frail<br>6m: non-frail<br>12m: death	Intervention	No admission recorded	N/a	B: 13.7<br>P4: 11*<br>P5: 12.2<br>P6: 11.7*	B: 26<br>P1: 22*<br>P3: 25	Prochlorperazine (used long term)
8	B: non frail<br>6m: non-frail<br>12m: death	Intervention	No admission recorded	N/a	B: 11.7<br>P1: 10.7<br>P2: 7.9*<br>P3: 13.4<br>P4: 11	B: 19<br>P1: 21 P3: 21	Denosumab 6 monthly
9	B: non frail<br>6m: non-frail<br>12m: death	Control	Unknown	Two days after admission	B: 16.4<br>6m: 11.8*	B: 28<br>6m: 26	Furosemide
10	B: non frail<br>6m: non-frail<br>12m: death	Control	Laparotomy (planned admission) - bowel cancer	1.5 months after surgery and discharge	B: 22.4<br>6m: 23.7	B: 28<br>6m: 30 (14/11/19)	Paracetamol

11	B: non frail<br>6m: non-frail<br>12m: frail	Control	Fall and broken right humerus	-	B: 20<br>6m: 15.8*<br>12m: 14.8*	B: 17<br>6m: 18<br>12m: 13*	Denosumab 6 monthly. Fourteen medicines added within 12 months including apixaban, frusemide and metoprolol as regular medicines and oxycodone and temazepam as prn
12	B: non frail<br>6m: non-frail<br>12m: frail	Control	No admission recorded	-	B: 7.7<br>6m: 10.1<br>12m: 8.8	B: 20<br>6m: 16*<br>12m: 13*	Oxycodone and naloxone

* Clinically significant deterioration
B: Baseline; 6m: 6 months; 12m: 12 months; MoCA: Montreal Cognitive Assessment (higher score indicates better cognitive function); N/a: Not available; P: Pharmacist intervention session

Table S2. Participants who transitioned from non-frail at baseline to frail at 6 months, and transitioned back to pre-frail at 12 months.

ID	Patient status at baseline, 6m and 12m	Trial arm	Primary Reason of hospital admission	Death after admission	Grip strength	Cognition (MoCA)	Medication change
13	B: non frail<br>6m: frail<br>12m: pre-frail	Intervention	Fall, infection	-	B: 10.8<br>P1: 10.7	B: 26<br>P1: 26	Domperidone (initiated five days before fall; used long-term)
14	B: non frail<br>6m: frail<br>12m: pre-frail	Intervention	No admission recorded	-	B: 14.3<br>P4: 11.4*	B: 24<br>P4: 23	Tramadol 50mg bd, reduced from 100mg bd)
15	B: non frail<br>6m: frail<br>12m: pre-frail	Control	No admission recorded	-	B: 8.9<br>6m: 13.6<br>12m: 14.1	B: 20<br>6m: 16*<br>12m: 14*	N/a

* Clinically significant deterioration
B: Baseline; 6m: 6 months; 12m: 12 months; MoCA: Montreal Cognitive Assessment (higher score indicates better cognitive function); N/a: Not available; P: Pharmacist intervention session
